# Under fire-simultaneous volatilome and transcriptome analysis unravels fine-scale responses of tansy chemotypes to dual herbivore attack

**DOI:** 10.1186/s12870-020-02745-1

**Published:** 2020-12-09

**Authors:** Mary V. Clancy, Georg Haberer, Werner Jud, Bishu Niederbacher, Simon Niederbacher, Matthias Senft, Sharon E. Zytynska, Wolfgang W. Weisser, Jörg-Peter Schnitzler

**Affiliations:** 1 Helmholtz Zentrum München, Research Unit Environmental Simulation (EUS), Institute of Biochemical Plant Pathology, Neuherberg, Germany; 2 Fundamental and Applied Research in Chemical Ecology (FARCE Lab), Institute of Biology, University of Neuchâtel, Neuchâtel, Switzerland; 3 Helmholtz Zentrum München, Plant Genome and Systems Biology, Neuherberg, Germany; 4 Terrestrial Ecology Research Group, Department of Ecology and Ecosystem Management, Technical University of Munich, School of Life Sciences Weihenstephan, Freising, Germany; 5 Department of Ecology, University of Liverpool, Evolution and Behaviour, Institute of Infection, Veterinary and Ecological Sciences, Liverpool, UK

## Abstract

**Background:**

Tansy plants (*Tanacetum vulgare* L.) are known for their high intraspecific chemical variation, especially of volatile organic compounds (VOC) from the terpenoid compound group. These VOCs are closely involved in plant-insect interactions and, when profiled, can be used to classify plants into groups known as chemotypes. Tansy chemotypes have been shown to influence plant-aphid interactions, however, to date no information is available on the response of different tansy chemotypes to simultaneous herbivory by more than one insect species.

**Results:**

Using a multi-cuvette system, we investigated the responses of five tansy chemotypes to feeding by sucking and/or chewing herbivores (aphids and caterpillars; *Metopeurum fuscoviride* Stroyan and *Spodoptera littoralis* Boisduval). Herbivory by caterpillars following aphid infestation led to a plant chemotype-specific change in the patterns of terpenoids stored in trichome hairs and in VOC emissions. The transcriptomic analysis of a plant chemotype represents the first de novo assembly of a transcriptome in tansy and demonstrates priming effects of aphids on a subsequent herbivory. Overall, we show that the five chemotypes do not react in the same way to the two herbivores. As expected, we found that caterpillar feeding increased VOC emissions, however, a priori aphid infestation only led to a further increase in VOC emissions for some chemotypes.

**Conclusions:**

We were able to show that different chemotypes respond to the double herbivore attack in different ways, and that pre-treatment with aphids had a priming effect on plants when they were subsequently exposed to a chewing herbivore. If neighbouring chemotypes in a field population react differently to herbivory/dual herbivory, this could possibly have effects from the individual level to the group level. Individuals of some chemotypes may respond more efficiently to herbivory stress than others, and in a group environment these “louder” chemotypes may affect the local insect community, including the natural enemies of herbivores, and other neighbouring plants.

**Supplementary Information:**

The online version contains supplementary material available at 10.1186/s12870-020-02745-1.

## Introduction

Nature is formed of complex communities in which plants play a central role. Interactions between plants and plant antagonists such as herbivores or pathogens result in ever-evolving defence mechanisms and corresponding efforts to overcome them [1]. Plants are sessile organisms, and as such have developed an arsenal of chemical defences in lieu of being able to flee. These plant secondary metabolites consist of diverse groups of compounds that vary considerably across species and families [2]. Different defence compounds challenge plant antagonists in different ways: direct defences include compounds that are actively detrimental to herbivores (for example digestibility reducers and toxins [3]), whereas indirect defences can function as a recruitment drive for attracting herbivore enemies [4]. One important group of compounds involved in indirect plant defence are volatile organic compounds (VOCs). Emission of VOCs by plants is often induced by herbivore attack on plants, but emissions can also be constitutive. VOCs are chemically highly diverse, also within plant species, and this can result in large variation in chemical profiles (i.e. chemotypes) both between and within plant populations [5, 6]. Terpenoids, in particular mono- and sesquiterpenoids, are the largest and most structurally diverse class of VOCs found in the plant kingdom. Terpenoids are naturally occurring organic compounds derived from terpenes. Most terpenoid compounds are multicyclic structures with oxygen-containing functional groups. Although they are sometimes referred to as “terpenes”, terpenoids contain additional functional groups, mostly containing oxygen [7]. The terpene synthase (TPS) gene family is responsible for this massive diversity of compounds, as a single TPS enzyme can catalyse up to 10 different structures from a single substrate [8, 9]. VOCs can be emitted immediately following damage caused by a herbivore, or can be induced and emitted several hours later [10, 11]. Traditional methods for assessing volatile emissions in plant-herbivore interactions continue to be improved upon, with more sensitive instruments coming to market [12, 13].

Many studies have investigated the effect of attack of single species of antagonist on plant volatile emissions [14] and have found that the response of plants depends on the type of damage that is inflicted. For instance, chewing herbivores such as caterpillars generally induce the jasmonic acid (JA) pathway (which works in synergy with the volatile phytohormone ethylene), whereas sucking herbivores such as aphids typically induce the salicylic acid (SA) pathway, which is most commonly associated with biotrophic pathogens [15]. Chewing herbivores can inflict significant damage to plant tissues, leading to large changes in metabolism [16]. The level of damage caused to a plant by a chewing herbivore is dependent on several factors, including larval stage, specialism/ generalism of plant-herbivore interaction, infestation threshold, and species [17]. In contrast, sucking herbivores such as aphids, cause a less obvious form of damage, as they suck on plant sap and deplete the plant of nutrients rather than destroying leaf tissue. However, wide-ranging induction of defence pathways in local and systemic leaves is also observed [18] for both chewing and sucking herbivores. Interestingly, interactions between the SA and JA pathways are usually antagonistic, although additive or synergistic responses have been reported as well [19]. These phytohormone-mediated defence pathways result in comprehensive changes to the metabolome, transcriptome, and proteome. In nature, plants are often attacked by several different antagonists, and more and more information is becoming available on the VOC response of plants attacked by multiple species of attacking herbivores or other antagonists [20].

An increasing number of studies shows that attack by one herbivore or pathogen can induce a state of heightened defence capabilities in plants [21]. Changes may occur at the transcriptional, physiological, and metabolic levels, which effectively enable the plant to react more quickly or strongly to biotic and abiotic stresses. This defence priming is mediated by a range of chemicals, such as salicylic acid and pipecolic acid [22], jasmonic acid and ethylene [23], azealic acid [24], and various volatile compounds including, methyl salicylate and methyl jasmonate [25], green leaf volatiles [26], and monoterpenes [27]. VOCs are increasingly shown to be important for defence priming and systemic responses. For example, rapid responses of plants to wounding include emissions of lipoxygenase pathway (LOX) products, known as green leaf volatiles, comprising of different C6 compounds such as hexenals and other aldehydes [28]. These compounds are produced and emitted within seconds of wounding stress and are typically transient, however emissions can be maintained for longer periods of time following repeated wounding or herbivory. VOCs are also involved in plant induced systemic immunity (known as systemic acquired resistance, or SAR) that is triggered throughout a plant after it has been exposed to a local pathogen infection. SAR is characterised by high levels of accumulated SA throughout the plant and induced volatile monoterpenes are essential for within-plant SAR [27], but it has recently been suggested that these compounds can also act as signalling molecules for long-distance SAR induction between plants [29]. As the defence pathways induced by different types of antagonists such as chewing and sucking herbivores can be antagonistic or synergistic, the question arises of how attack by both feeding guilds affects induction of chemical defences. While studies have already described complex interactions including defence priming, we are far from understanding the complex response of plants to attack by multiple herbivores.

*Tanacetum vulgare* L. (Asteraceae), commonly known as tansy, is a highly aromatic herb that has extensive variation in terpenoid content stored in glands on the leaf surface [30–32]. Tansy plants can be grouped according to their terpenoid content, forming chemotypes [6, 33]. Tansy chemotypes have been shown to significantly affect the colonisation of a specialised aphid species early in the season (during the main dispersal event when winged morphs are produced), however failed to have an effect on aphid colonisation later in the season, when aphids are unwinged and dispersal is via walking only [6]. One of these specialist aphids is *Metopeurum fuscoviride* Stroyan (Aphididae). Although *M. fuscoviride* is specialised to tansy, and causes minimally invasive damage, it can induce salicylic acid defence responses [34]. While tansy chemotypes affect aphid preference [35], and performance in the field [6, 34], it is unclear if plants of different chemotypes also differ in their response to aphid attack. As tansy is attacked not only by sucking herbivores but also chewing ones, chemotypes may differ in how aphid priming affects the plant’s response to subsequent attack by chewing herbivores.

In the present study we investigated how tansy chemotypes responded to attack by chewing herbivores when the plants had, or had not, been previously attacked by sucking aphids. VOCs can be emitted immediately following damage caused by a herbivore, or can be induced and emitted several hours later [10]. Here we looked at the effects of tansy chemotypes on the emission of VOCs triggered by two insect herbivores from differing feeding guilds; sucking aphids and chewing caterpillar larvae. To measure the real-time VOC emissions we used a platform introduced by Jud et al., 2018 [12], where we combine gas chromatography- mass spectroscopy (GC-MS) and proton transfer reaction time of flight mass spectroscopy (PTR-ToF-MS, [36]). We show that there are strong differences in VOC emissions upon herbivory across chemotypes. Furthermore, using RNA-Seq and de novo assembly of transcriptomes we examined transcriptional changes, i.e. induced/suppressed gene expression following aphid and caterpillar herbivory, with a view to simultaneously analyse metabolomic and transcriptomic changes to observe any inducible response of the plants to both sucking and chewing herbivores.

## Results

**Fig. 1:**
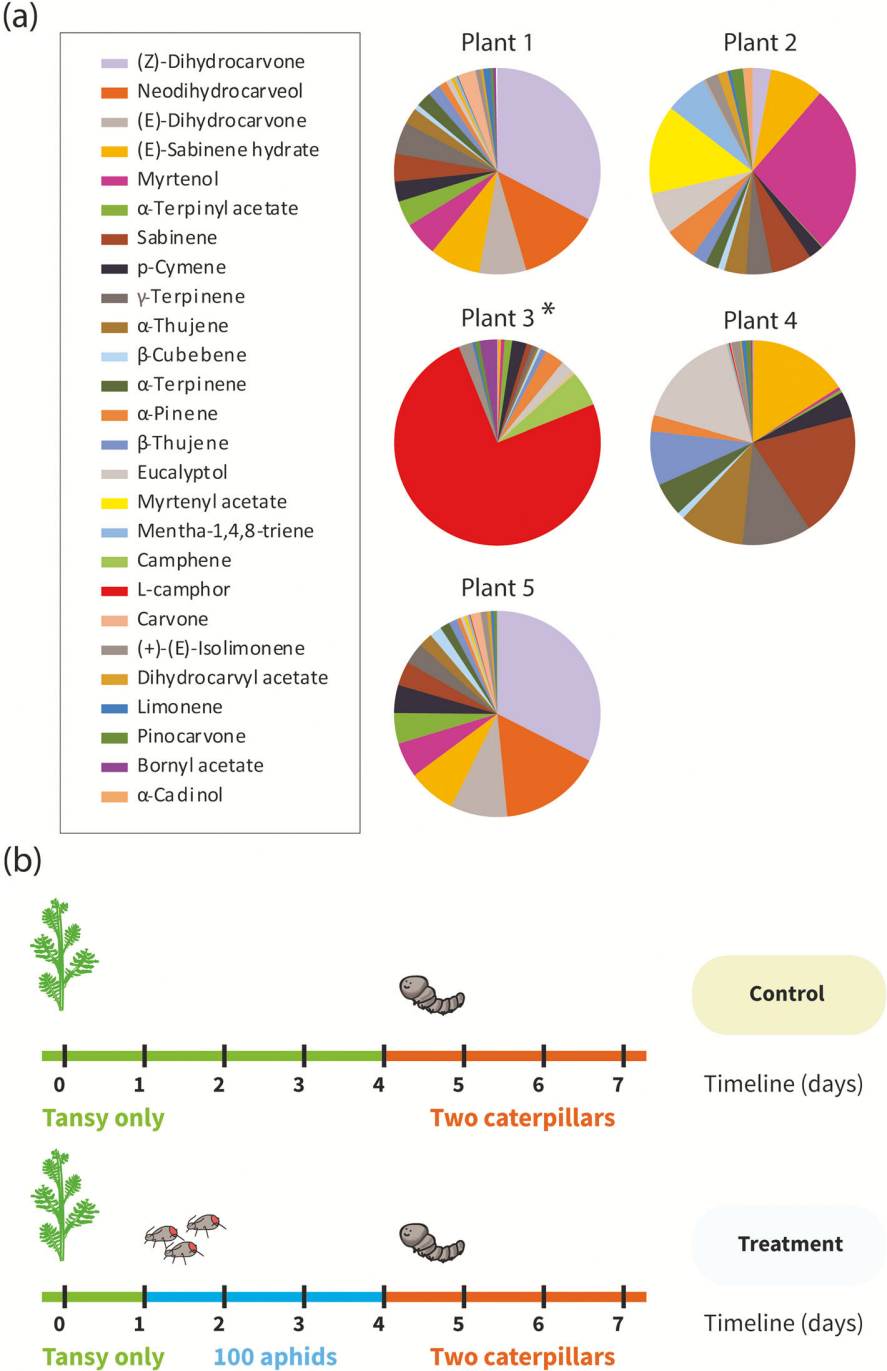
**a** chemotype groupings of plants used in the cuvette experiment. Plant 3 was used for transcriptome analysis; **b** schematic of experimental timeline. Leaf material for RNA and liquid extraction was harvested on days 4 and 7. Leaf volatile emissions were continuously measured using PTR-ToF-MS, and collected daily using adsorbent Tenax and Carbopack cartridges

In the central experiment of the work (Fig. 1b), the five terpenoid chemotypes were cultivated in a multi cuvette system (the scheme of the system is depicted in the additional Fig. S1) over seven days, continuously measuring the VOC emissions. At certain timepoints (days 4 and 7) leaf samples were also taken from all plants for chemical analyses, and the transcriptome study (only of plant chemotype 3). In order to select plants containing a wide range of compounds, we assessed the VOC profile of eight tansy plants using the hexane extraction method outlined in [6]. A total of 48 compounds were identified, and five chemotypes were assigned according to relative dominance of compounds (Fig. 1a). All plant chemotypes were propagated by splitting plants into nine daughter clones, which were further used as biological replicates as the chemotype is stable among clones [6].

### Changes of terpenoid patterns in the storage pools of tansy chemotypes caused by aphids and caterpillars (hexane extractions)

**Fig. 2:**
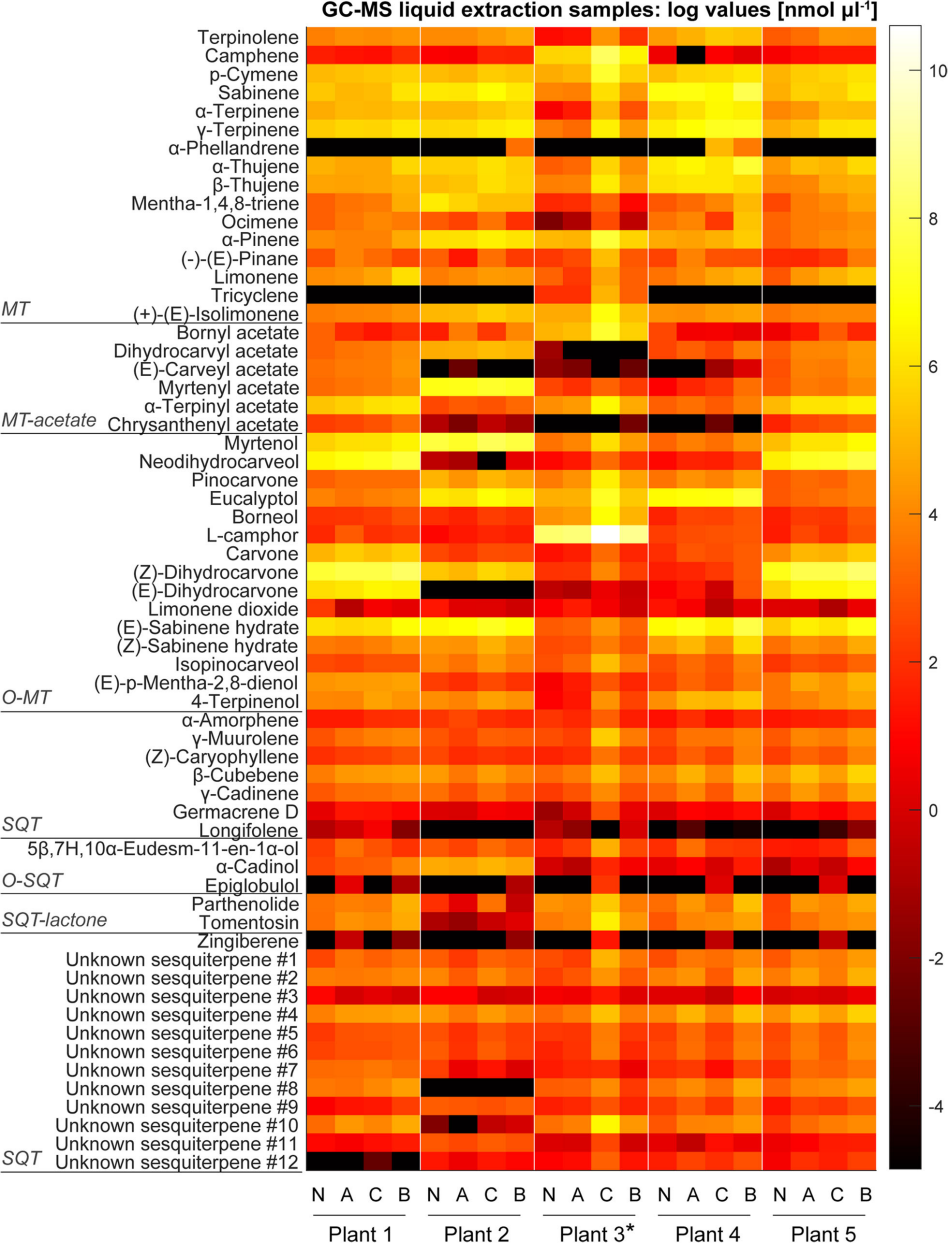
Heatmap showing changes in concentration of VOCs measured using the hexane extraction method after each treatment. Each point in the heatmap represents the averaged data from three biological replicates (*n* = 3 for each treatment) and two technical replicates. N: no aphid, no caterpillar, leaf material harvested on day 4; A: aphid, no caterpillar, leaf material harvested on day 7; C: no aphid, caterpillar, leaf material harvested on day 4; B: both aphid and caterpillar, leaf material harvested on day 7. The VOC concentrations for the heatmap are listed in supplemental Table S2. *Plant 3 was used for transcriptome analysis. MT: monoterpene, MT-acetate: monoterpene acetate, O-MT: oxygenated monoterpene, SQT: sesquiterpene, O-SQT: oxygenated sesquiterpene, SQT-lactone: sesquiterpene lactone

A total of 64 compounds were identified across all chemotypes using GC-MS analysis of the hexane extracts, which were grouped into six compound classes (see Table S1). The compounds detected were mostly mono- and sesquiterpenes; the increased number of identified compounds in comparison to the initial chemotyping is due to higher overall emissions and herbivory induced stress compounds. Plant chemotypes 1 and 5 were partially dominated by (*Z*)-dihydrocarvone (> 30%), while plant chemotype 2 was characterised by a slight dominance of myrtenol (~ 25%) followed by myrtenyl acetate (> 10%). Plant chemotype 3 was strongly dominated by L-camphor (> 70%), whereas plant chemotype 4 was slightly dominated by sabinene (~ 20%), with eucalyptol (~ 15%) and (E)-sabinene hydrate (> 10%) also found in the mixture (Fig. 1a). Figure 2 shows clear differences between the five chemotypes.

A two-tailed t-test analysis on the summed concentrations of all volatiles across plant chemotypes per treatment revealed no significant differences between treatment groups except between treatment groups N and B (both aphids and caterpillar; t-value = − 3.659, df = 4, *P* = 0.022; Fig. S2). While there were little differences in total emission, there were significant differences among chemotypes in their response to herbivory of the two different species.

The terpenoid pattern of treatment group N plants (no aphid, no caterpillar) in Fig. 2 represents the unstressed chemotype, with correspondingly lower concentrations of terpenoids detected in the hexane extracts compared to the other treatments (compound concentrations are listed Table S2). Treatment group A plants (aphid, no caterpillar) show the influence of aphid feeding on the profile of the stored (hexane extracted terpenoid compounds) chemotype. The aphid effect on the pattern and concentration of stored terpenoids was minimal; in plant chemotypes 1, 3, 4, and 5 the concentrations of terpenoids increased slightly, whereas in the chemotype 2 the concentrations were reduced.

While aphids are minimally invasive phloem suckers, and as such do not cause a great physically apparent stress on the plant, caterpillars are chewing herbivores and can cause severe tissue loss, resulting in huge physical trauma to the plant. Treatment group C plants (no aphid, caterpillar) show a varied response to caterpillar damage; with plant chemotypes 2 and 3 exhibiting a strong increase in terpenoid concentration. In plant chemotypes 1 and 5 very little increase in VOC concentration could be observed in treatment group C, whereas plant chemotype 4 appeared to show a decrease in terpenoid accumulation across all compounds apart from eucalyptol, the concentration of which was slightly increased. Treatment group B plants (both aphid and caterpillar) showed markedly increased concentrations of terpenoids in all chemotypes except in plant chemotypes 2 and 3.

**Fig. 3:**
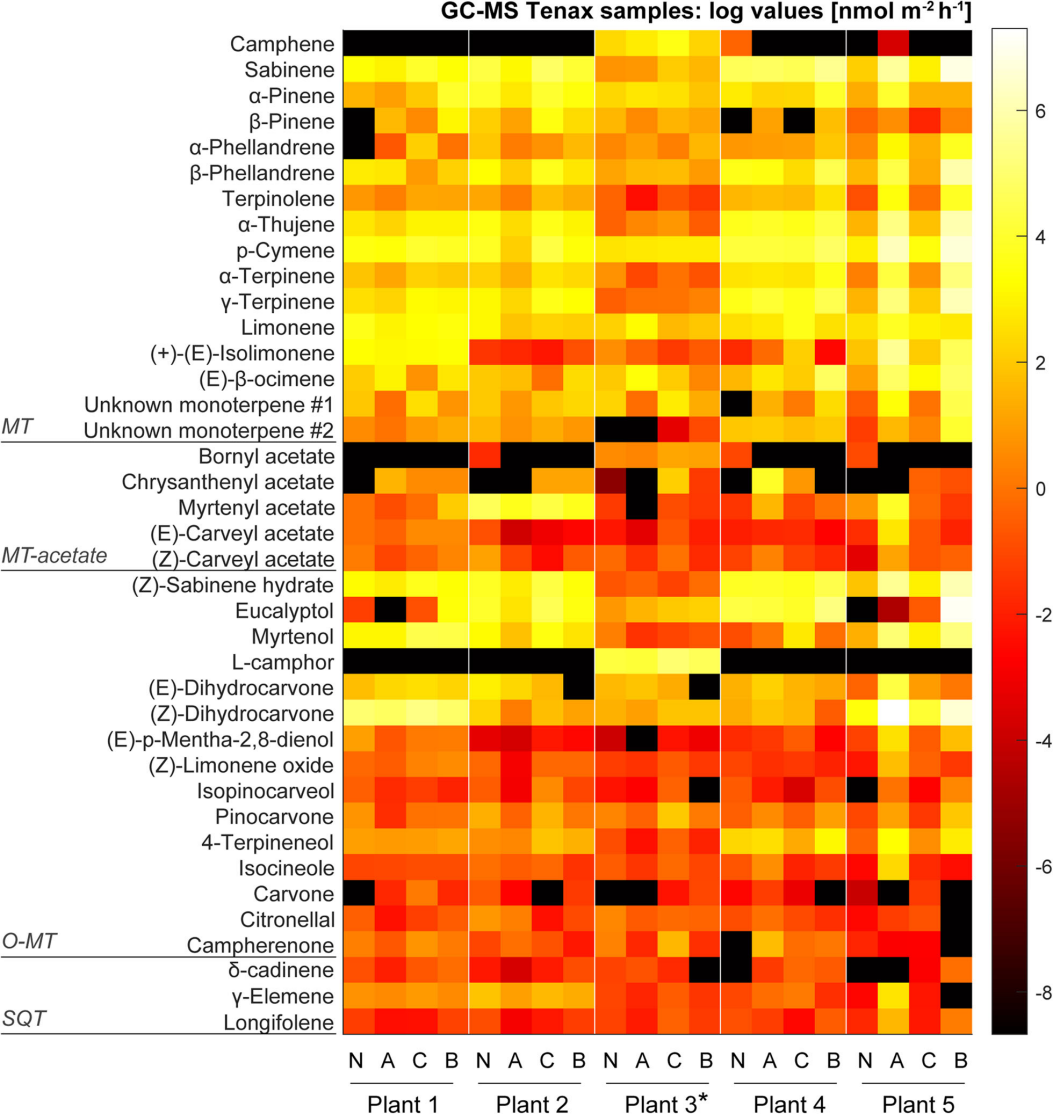
Heatmap showing changes in VOC emissions (logarithmic scale) after treatments using headspace analysis. VOCs were focused on Tenax/ Carbopack cartridges. Each point in the heatmap represents the averaged data from three biological replicates (*n* = 3 for each treatment. N: no aphid, no caterpillar, average of data from days 1–4; A: aphid, no caterpillar, average of data from days 5–7; C: no aphid, caterpillar, average of data from days 1–4; B: both aphid and caterpillar, average of data from days 5–7. The VOC emission rates for the heatmap are listed in supplemental Table S3. *Plant 3 was used for transcriptome analysis. MT: monoterpene, MT-acetate: monoterpene acetate, O-MT: oxygenated monoterpene, SQT: sesquiterpene, O-SQT: oxygenated sesquiterpene. Compound concentrations can be found in Table S3

The high concentration of detected terpenoids (in particular L-camphor) found in plant chemotype 3 after application of caterpillars (treatment group C) is reflected in the VOC emission pattern of this chemotype (Fig. 3). While the hexane extraction method gives an overview of all compounds that are synthesised and stored within the leaf structures, the collection of VOCs on Tenax/ Carbopack cartridges from the cuvette outlet air comprises only compounds that were actually released from the plant into the headspace of the cuvettes. The confirmation of high levels of VOCs detected in the headspace samples (see next section) were in line with the results obtained from the hexane extracts. This indicates that these compounds were synthesised and stored/released in response to herbivore feeding.

### Emission pattern of terpenoids following aphid and caterpillar herbivory (volatile measurements from filters)

To obtain an overview of the compounds emitted by each plant chemotype over a specific timeframe, emitted VOCs were collected on Tenax/ Carbopack cartridges for a period of 2–3 h (see methods). Forty compounds were identified by GC-MS, and are listed in supplemental Table S1. Compounds found in the headspace analysis were mainly categorised as mono- and sesquiterpenoids. Emission patterns were clearly defined by the chemotype grouping (Fig. 3, supplemental Table S2), and were similar in composition to those seen in the initial chemotyping using the liquid extraction method. A main result was that responses of tansy to herbivore attack depended on plant chemotype.

Emission of plant chemotype 1 was dominated by (*Z*)-dihydrocarvone. Plants treated with caterpillars (treatment group C; no aphids, caterpillars) showed an increase in myrtenol and p-cymene emissions. Plant chemotype 2 was slightly dominated by sabinene and myrtenyl acetate. Plant chemotype 3 was strongly dominated by L-camphor. Plant chemotypes 1, 2, and 3 belonging to the treatment group C (no aphids, caterpillars) all emitted slightly higher levels of VOCs than plants pre-treated with aphids. Plant chemotype 4 emitted a more even blend of compounds with a slight dominance of sabinene, and eucalyptol. (*Z*)-dihydrocarvone was strongly emitted by plant chemotype 5, with a variety of other compounds including p-cymene and sabinene. In contrast, plant chemotypes 4 and 5 that were pre-treated with aphids (treatment group B) displayed higher VOC emissions than untreated plants (treatment group C). The emission patterns observed in the Tenax/ Carbopack measurements (Fig. 3) were also reflected in both the hexane extracts (Fig. 2) and PTR-ToF-MS analyses (see next section).

### Dynamic emissions of tansy volatiles during aphid and caterpillar feeding (continuous volatile emission measurements)

Tansy VOC emissions were continuously measured online using PTR-ToF-MS both before and after application of aphids and caterpillars. The time course of emissions demonstrated the effects of herbivore damage across all five chemotypes, and again pointed to chemotype-specific reactions to herbivore attack.

**Fig. 4:**
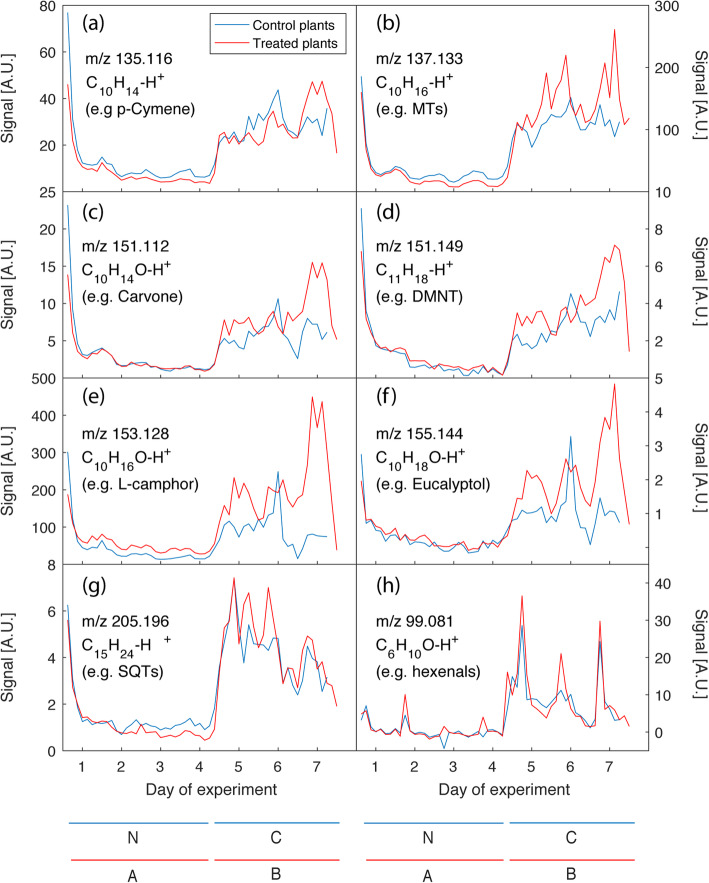
Time course of selected VOC emissions measured online using PTR-ToF-MS. Emission data from all chemotypes was averaged according to treatment. Plants that did not receive aphid treatment are represented by the blue lines. Plants that received aphid treatment are represented by the red lines. Where applicable, aphids were applied on day 1; caterpillars were applied on day 4. Leaf biomass was harvested on days 4 and 7. N: no aphid, no caterpillar, A: aphid, no caterpillar, C: no aphid, caterpillar, B: both aphid and caterpillar. **a** *m/z* 135.116, representing monoterpenoids such as p-cymene; **b** *m/z* 137.133, representing monoterpenes such as limonene; **c** *m/z* 151.112, representing terpenoids such as carvone; **d** m/z 151.149, representing volatile compounds such as (*E*)-4,8-dimethyl-1,3,7-nonatriene (DMNT); **e** *m/z* 153.128, representing oxygenated monoterpenoids such as L-camphor; **f** *m/z* 155.144, representing oxygenated monoterpenoids such as eucalyptol; **g** *m/z* 205.196, representing sesquiterpenoids such as germacrene D; **h** *m/z* 99.081, representing hexenals, also known as green leaf volatiles (GLVs)

Diurnal variation in terpenoid emissions was observed across all plant chemotypes (e.g. sum of monoterpenes at *m/z* 137.133 and sesquiterpenes at *m/z* 205.196; see Fig. 4b and g respectively). High levels of emissions were seen at the onset of the experiment, when the plants were placed in the cuvettes. Except hexenal (mass *m/z* 99.081), the emission rates of all detectable mass features were elevated at the beginning of the measurements and declined within the first 24 h. This indicates that no mechanical damage to the plants occurred when the plants were placed in the cuvettes. Emissions from the first 24 h were not included in any data analysis as they were scattered and not representative of baseline volatile emissions from unstressed plants.

**Fig. 5:**
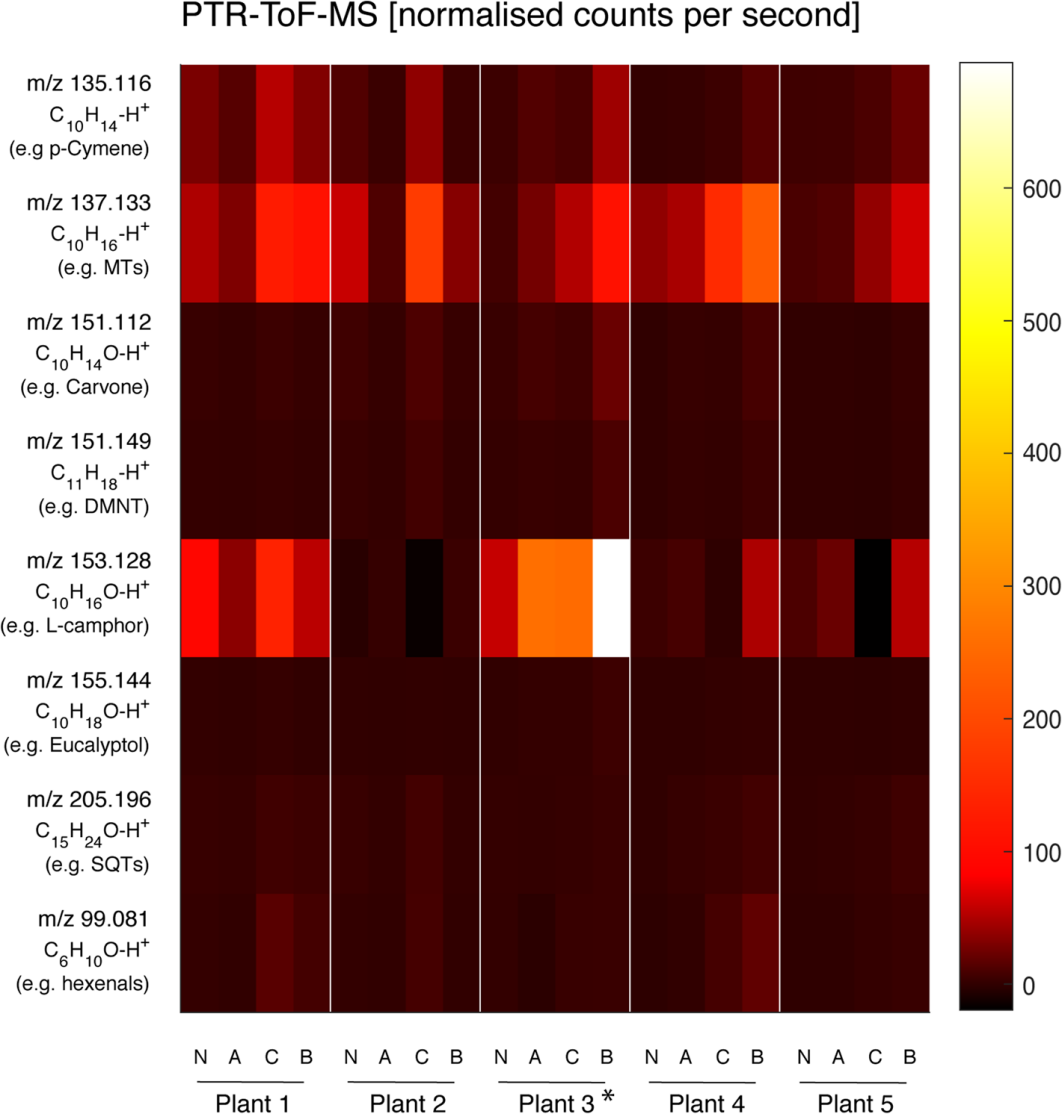
Heatmap showing changes in emissions of representative masses measured using PTR-ToF-MS. Each point in the heatmap represents the averaged data from three biological replicates (*n* = 3 for each treatment group). For comparison with the off-line GC-Ms analysis of adsorbent cartridges (0.1 L min^− 1^; collection period of 3 h around noon), PTR-ToF-MS data were averaged for the same time intervals. N: no aphid, no caterpillar, average of data from days 1–4; A: aphid, no caterpillar, average of data from days 5–7; C: no aphid, caterpillar, average of data from days 1–4; B: both aphid and caterpillar, average of data from days 5–7. The VOC emission rates for the heatmap are listed in supplemental Table S5. *Plant 3 was used for transcriptome analysis

Application of aphids had no significant effect on the overall emission rates of the different compounds detected by PTR-ToF-MS. No obvious increase in emission occurred until day four, except a diurnal variation of the sum of monoterpenes (MTs; *m/z* 137.133; Fig. 4b) and three oxygenated monoterpenoids (O-MTs; *m/z* 135.116, *m/z* 151.112, *m/z* 153.128; see Fig. 4a, c, and e respectively; Supplemental Table S3). Addition of caterpillar larvae immediately changed tansy emissions, with all emission rates immediately increasing, whereas the diurnal variations can be seen in some mass features. It is not clear from the profiles whether the increase in emissions is merely a wounding effect or a rapid induction of the emissions after application of the chewing herbivores. A transient increase in the emission of dimethylnonatriene (DMNT, *m/z* 151.149; Fig. 4d), MTs, and the O-MTs was observed. In contrast, the emission of sesquiterpenes (SQTs; *m/z* 205.196) decreased after a sharp increase over time, while the emission of green leaf volatiles, exemplified by the hexenal signal at m/z 99.081, Fig. 4h) showed an initial increase, followed by a strong diurnal fluctuation, which did not increase over time. Plants that received aphid treatment first showed an overall tendency towards higher emission rates of MTs and O-MTs as well as the C11 homoterpene DMNT compared to plants that were not pre-treated by the sucking insects (Fig. 5).

A heatmap analysis (Fig. 5, supplemental Table S4) of the averaged on-line mass spectrometric data visually confirms the classification of the plant chemotypes done by GC-MS analysis of the hexane extracts (Fig. 2). For comparison with the off-line GC-MS analysis of VOCs collected on adsorbent cartridges (0.1 L min^− 1^; collection period of 3 h around noon), PTR-ToF-MS data from the same time intervals were averaged. Treatment group N is the average of data collected from days 1 to 4 of plants that were not infested with aphids, while treatment group A is the average of the same time but of plants that were infested with aphids. Treatment group C is the average of data collected from days 4 to 7 of plants that were subjected to caterpillar feeding but not aphid infestation, while treatment group B is the average of the same time but of plants that were subjected to feeding by both aphids and caterpillars. Chemotypic profiles of the five plants are highlighted by the differences in signal strength between MTs (*m/z* 137.133; e.g. sabinene and γ-terpinene) and O-MTs (*m/z* 153.128; e.g. L-camphor and (*Z*)-dihydrocarvone). After the addition of aphids to plant chemotypes 1 and 2, a decrease in MT and O-MT levels could be detected, whereas plant chemotypes 3, 4, and 5 showed an increase in these monoterpenoids. Each chemotype responded differently to the aphid treatment. While caterpillar feeding generally increased plant VOC emissions, prior feeding by aphids further increased this for plant chemotypes 3, 4, and 5, but reduced it for chemotypes 1 and 2.

### Transcriptome changes in chemotype 3 following aphid and caterpillar feeding

Following RNA-Seq analysis of RNA extracted from plant chemotype 3, de novo assembly yielded a total of 52,765 plant genes that were surveyed for transcriptional changes resulting from the combinations of aphid and caterpillar treatments. Differences between treatment contrast groups are outlined as follows: A-N = aphid effect, no caterpillar (*a*), C-N = caterpillar effect, no aphid (*c*), B-A = caterpillar effect, with aphid (*ca*), (B-A) – (C-N) = aphid effect, with caterpillar (*d*) (see Fig. S3).

**Fig. 6:**
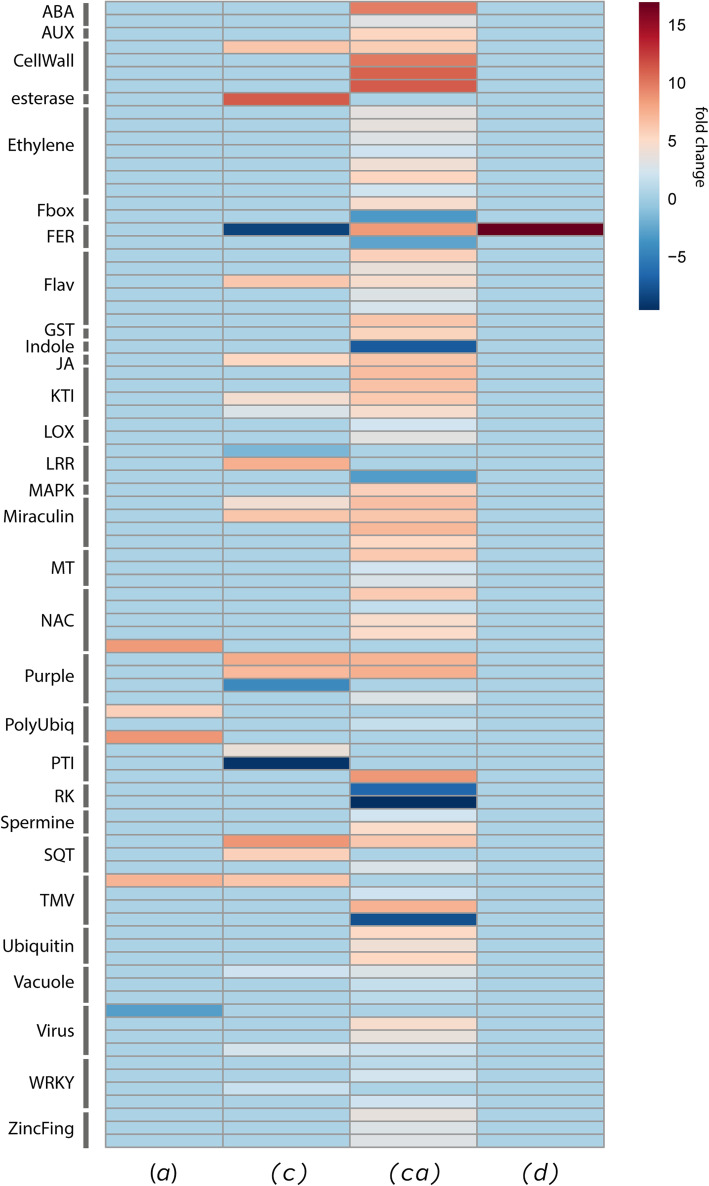
Heatmap visually representing differentially expressed genes related to defence processes from plant 3. Column A shows the effect of aphids with no caterpillars vs no aphids and no caterpillars (treatment groups A-N), C shows the caterpillar effect with no aphids vs no aphids and no caterpillars (treatment groups C-N), (*ca*) shows the effect of caterpillars with aphids vs caterpillars and no aphids (treatment groups B-A), and D shows the effects of caterpillars with aphids vs caterpillars with no aphids (treatment groups (B-A)-(C-N))

A total of 502 differentially expressed genes (DEGs) were found with a fold change greater than two whether up- or down-regulated (*P* < 0.05; supplemental Table S5). DEGs associated with various defence responses are detailed in supplemental Table S6 and visualised in a heatmap in Fig. 6. Consistent with the metabolomics data above, the aphid treatment contrast (*a*) elicited only a minor transcriptional response. DEGs encoding a putative NAC transcription factor 56 (TanvuEGr019790, ortholog to AT2G41890.1, *Arabidopsis thaliana*; E value: 2.39e-17; 8.5 fold-change) and tobacco mosaic virus (TMV) resistance protein N (TanvuEGr041015, ortholog to OIT35319, *Nicotiana attenuata*; E-value: 2.6e-10; 7.3 fold-change) were upregulated. Considerably larger effects were observed for the infestation of plants with caterpillars. In treatment group contrasts involving caterpillar treatment, a series of Trypsin/chymotrypsin inhibitors orthologs TanvuEGr038583 (ortholog to AT1G73325, *A. thaliana*; E-value: 1.1e-3) TanvuEGr040924 (ortholog to AT1G73325, *A. thaliana*; E-value: 2.3e-5) TanvuEGr035344 (ortholog to AT1G73325, *A. thaliana*; E-value: 2.1e-3)) and putative abrin- and nigrin-like genes (impeding protein biosynthesis) were upregulated. Several genes were differentially regulated following caterpillar feeding only; a lipase (TanvuEGr016806, ortholog to AT3G04290.1, *A. thaliana*; E-value: 1.35e-4) was strongly upregulated (11.2 fold-change), while the receptor-like protein kinase FERONIA gene (TanvuEGr013486 (ortholog to AT3G51550.1, *A. thaliana*; E-value: 1.19e-2) was downregulated (− 8.5 fold-change). Plants that were treated with both caterpillars and aphids (treatment contrast group (*ca*)) had the highest number of DEGs out of the four analysed treatment contrast groups. DEGs associated with cell wall processes including putative endochitinase EP3 (TanvuEGr027756, ortholog to AT3G54420.1, *A. thaliana*; E-value: 1.12e-11) and laccase-7 (TanvuEGr007097, ortholog to AT3G09220.1, *A. thaliana*; E-value: 1.28e-03) genes were strongly upregulated (10 and 11.2 fold-change, respectively). The same FERONIA gene, shown to be downregulated by caterpillar treatment only, was under the combined treatment highly upregulated (8.5 fold-change). An ortholog to AT4G27260 (*A. thaliana*; E-value: ~ 0), Indole-3-acetic acid-amido synthetase GH3.5 (TanvuEGr005980) was strongly downregulated (− 7.1 fold-change), as were two putative G-type lectin S-receptor-like serine/threonine-protein kinases, SD2–5 (TanvuEGr003778, ortholog to AT4G32300.1, *A. thaliana*; E-value: 2.64e-02) and SD3–1 (TanvuEGr002669, ortholog to AT2G41890.1, *A. thaliana*; E-value: 2.39e-17) (− 6.5 and − 9.6 fold-change respectively). Plants that were treated with both aphids and caterpillars, relative to plants that received only caterpillars (treatment contrast group (*d*)) did not exhibit many DEGs, however again the FERONIA gene mentioned before was very strongly upregulated (16.9 fold-change).

### Phylogenetic analysis of TPSs from tansy and other plant species

Since mono- and sesquiterpenes are basic components of the essential oils in the trichomes and they also dominate the volatile emissions of tansy, it is interesting to study the expression of the gene family on terpene synthases. The sequences of *TPS* genes from closely related plant species such as *Helianthus annuus* and *Artemisia annua*, were retrieved from online databases and aligned with the sequences of putatively annotated *TPS* genes from tansy. Phylogenetic analysis indicates that the tansy *TPSs* belong to the *TPS* subfamilies predicted (Fig. S4); for example, a putative (*E*)-β-ocimene synthase (TanvuEGr006575, ortholog to PWA70010.1, *A. annua*; E-value: 4e-147) and a putative sesquiterpene cyclase (TanvuEGr007220, ortholog to AAG24640.2, *A. annua*, E-value: 1e-124). The RNA-Seq data, however show that gene transcripts of putative *TPSs* are present, but only few show a change in their gene expression (Fig. 6, supplemental Table S5), e.g. two putative limonoid UDP-glucosyltransferases (TanvuEGr011661, (ortholog to AT4G15480.1, *A. thaliana*; E-value: 1.26e-04) and TanvuEGr028241 (ortholog to AT4G15480.1, *A. thaliana*; E-value: 3.49e-03)) and three *SQT* genes: a putative (−)-germacrene D synthase (TanvuEGr017925, ortholog to AT3G14490.1, *A. thaliana*; E-value: 2.73e-08) and two genes (TanvuEGr029614 and TanvuEGr007220) that are close orthologs to (*E*)-β-farnesene synthase (AT5G23960.1, *A. thaliana*; E-values: 1.02e-03 and 2.37e-03 respectively). While TanvuEGr029614 was upregulated (5.7 fold-change) under aphid treatment, the other a putative (*E*)-β-farnesene synthase only showed enhanced transcript level (2.8 fold-change) following aphid feeding and caterpillar attack.

## Discussion

We combined a number of methods, lead extracts, cumulative headspace sampling and real-time headspace sampling using a multi-cuvette system [12], to investigate the response of five different tansy chemotypes to attack by a sucking and a chewing herbivore, and priming effects. Aphid feeding did not result in strong chemical changes, which might be related to a minimum infestation threshold of feeding aphids [37], whereas caterpillar feeding increased stored and emitted volatiles across each plant chemotype. Importantly, there were chemotype-specific responses to herbivore attack. We also found evidence for priming, but the strength was dependent on the chemotype. Feeding by aphids followed by caterpillars led to more varied effects on stored and emitted volatile compounds in each plant chemotype than if aphids had not been feeding on the plant before a caterpillar was introduced. Specifically, prior aphid feeding further increased concentrations of stored volatile compounds in plant chemotypes 1, 4, and 5, and increased concentrations of compounds emitted to the headspace in plant chemotypes 3, 4, and 5. We also demonstrated that the time point of measurements affects the results obtained, particularly in relation to plant handling and insect feeding patterns. Transcriptomic analysis of plant chemotype 3 represents the first de novo assembly of a tansy transcriptome.

### Differences in responses to aphids and caterpillars: aphids

In this study, the different feeding mechanisms of the herbivores elicited very different defence responses in the tansy plants. Aphid feeding has been shown to exhibit variable effects on plant VOC emissions both alone and in conjunction with other herbivores. Schwartzberg and colleagues reported that exposure to the pea aphid (*Acyrthosiphon pisum*) did not induce any detectable changes in VOC emissions in its host plant *Vicia faba* [38], however contrastingly Du and colleagues showed that feeding by *A. pisum* on *V. faba* resulted in the induction and/or emission of several compounds [39]. Interestingly, Staudt and colleagues [40] found quantitative and qualitative differences in VOC emissions in peach cultivars (*Prunus* spp) exposed to *Myzus persicae*, with these differences being genotype-dependent. *Metopeurum fuscoviride* is specialised to tansy and may have co-evolved with the plant to minimise negative effects on each other, or the aphid may have evolved to avoid tansy defences, which could explain the minimal change in VOC emissions observed. It must be kept in mind that the intensity of aphid attack is also a key factor with aphid-induced volatiles; a minimum threshold of infestation of feeding aphids must be reached. Our previous work showed that aphid selection of host plants is non-random; early in the season aphids actively chose their host plant and colonised preferred chemotypes [6], and late in the season had almost exclusively colonised plants belonging to preferred metabotypes (irrespective of chemotype) [34]. Although aphids might select a host plant based on the volatile chemotype, the probability that they will successfully establish and maintain a colony is higher on certain metabotypes, and is related to other factors such as colony size and the presence of tending ants.

### Differences in responses to aphids and caterpillars: caterpillars and priming

As expected, plants responded very strongly to caterpillar attack and an increase in monoterpene emissions was observed in every chemotype after the caterpillars were applied at day 4. We also found evidence of priming: the overall trend showed that the production of monoterpenes was highest in plants treated with both aphids and caterpillars, indicating an effect of the aphids on terpenoid biosynthesis. Measured in the lipophilic hexane extracts, the change in synthesis / storage of monoterpenes (compounds we measured, not including all known compounds often released following aphid feeding) after application of aphids is minimal, being slightly decreased in relation to the control (treatment group N) in plant chemotype 2, slightly increased in plant chemotypes 1, 4, and 5, and seemingly unchanged in plant chemotypes 3. Thus, chemotypes showed higher or lower emissions depending on whether they had been pre-treated with aphids. In plant chemotypes 2 and 3, the volatile response was higher in plants that were not treated with aphids, whereas plant chemotypes 1, 4, and 5 displayed a stronger volatile response when they were pre-treated with aphids., When all volatiles (measured in the hexane extracts) across all plants per treatment were summed, no significant differences were observed except between treatment groups N (no attack by any herbivore) and B (attack by both herbivores), showing that it is not the absolute amount of volatiles that differed but rather changes in the composition.

Different priming effects after aphid feeding have also been observed in other studies. Schwarzberg and colleagues [38] found that emission of caterpillar-induced VOCs was reduced when the plants were co-infested with aphids. Conversely, other studies have found that aphid feeding increases total emissions of volatile terpenoids when subsequently attacked by species of different feeding guilds [41, 42]. Although there are differences in the experimental systems and specificity of aphid species, our observation that each chemotype responded differently to the herbivory treatments emphasises that there are chemo−/genotypic responses of plants to multiple herbivore attack.

### PTR-ToF-MS and other methods points to disruption of trichomes

The use of PTR-ToF-MS allowed us to observe changes in VOC emissions in real time. Increases in emissions of terpenoid compounds upon exposure to caterpillars were seen at random times of the day and night, likely reflecting activity patterns of feeding caterpillars. Caterpillars generally consume all parts of the leaf tissue and consequently will disrupt glandular trichomes while feeding. Due to this is was not possible to discriminate whether compounds were released from trichomes, i.e. were stored, or originated from de novo biosynthesis of tansy plants. However, the strong fluctuations in the emissions and pronounced peaks suggest that the destruction of trichomes and the resulting immediate evaporation of VOCs is a major cause of the emission. Emission courses of plants without trichomes usually show stable, homogeneous emissions of terpenoids when under herbivore feeding attack [43].

The interpretation is reinforced by the observation that volatile emission composition patterns of the different chemotypes were not drastically changed by the application of the two herbivores, but instead the levels of emission of dominant compounds and other closely related compounds increased. This suggests that the destruction or mechanical/physical stress of the trichome hairs by the arthropod movements contributed significantly to the increase in VOC emissions. Closely related compounds such as myrtenol and myrtenyl acetate, or dihydrocarvone and neodihydrocarveol, tended to all increase simultaneously. This also implies that herbivores feeding on different chemotypes causes different volatile profiles, rather than always the same.

### Local versus overall responses

Local and overall (systemic) volatile responses to both types of herbivory were investigated using two collection methods for GC-MS analysis. Hexane extraction, using control leaves and leaves from highly damaged sites, provided information on the local response to herbivory, while headspace analysis gave a view of the overall plant response. The high concentration of terpenoids (especially L-camphor) found in lipophilic extracts of plant chemotype 3 after aphid application is reflected in the emission patterns and rates of the plants. The increase in the concentration of stored terpenoids suggests an increased synthesis and storage of compounds, which then also contributes to an increased emission. In order to be able to clearly identify the origin of the emitted VOCs - from storage pools or from de novo biosynthesis - a ^13^C labelling experiment would have to be carried out (e.g. Ghirardo et al. 2020 [44]), however this was beyond the scope of this study.

### Gene expression, identification of genes, differential responses to herbivores

Our transcriptome analysis of plant chemotype 3 confirmed that several genes involved in terpenoid synthesis and defence were differentially regulated depending on the treatment. In the transcriptomic analysis, treatment contrast group (*a*), detailing plants that were treated with aphids in relation to untreated plants, DEGs encoding tansy orthologs of NAC56 transcription factor and a TMV resistance protein N were upregulated. The NAC56 transcription factor (TF) is known to play a role in plant responses to abiotic stress and challenge by pathogens [45]. Chen and colleagues demonstrated that in *Brassica napus* (oilseed rape), *BnNAC56* was significantly induced by JA, SA, and ABA, indicating that it may play a central role in plant tolerance of biotic stresses. Although there is limited evidence that NAC TFs regulate hypersensitive response (HR), this study suggests that BnaNAC56 induces HR-like cell death and thus is a form of inducible defence. TMV resistance protein N is a disease resistance protein that when triggered can induce a hypersensitive response [46]. It is interesting that DEGs commonly associated with pathogen infection were upregulated in tansy upon aphid feeding, as it is known that plants mount a similar defence against both aphids and biotrophic pathogens [47]. In treatment contrast group (*c*), detailing plants that were treated with caterpillars in relation to untreated plants, the putative receptor-like protein kinase *TvFERONIA* was strongly downregulated. In response to chewing herbivory, the JA defence pathway is induced. As FERONIA regulates JA signalling in an antagonistic way [48], it is unsurprising that it was downregulated in tansy plants that received only caterpillar treatment.

Plants that had experienced both herbivores (treatment contrast group (*ca*), detailing plants that were treated with aphids and caterpillars in relation to plants that received only aphid treatment) had the highest number of DEGs out of all four treatment groups. Tansy orthologs of endochitinase *EP3* and *laccase-7* were both strongly upregulated in these plants. It has been previously shown that overexpression of the cotton laccase gene led to higher resistance against both fungal plant pathogens (*Verticillium dahlia)* and herbivorous pests (cotton bollworm *Helicoverpa armigera and the cotton aphid Aphis gosypii)* in cotton plants [49]. Indole-3-acetic acid-amino synthetase *GH3.5* belongs to an auxin responsive gene family, and is responsible for catalysing the synthesis of indole-3-acetic acid (IAA). IAA helps plants deal with the presence of excess auxin, and overexpression of *GH3.5* results in enhanced resistance to fungal pathogens in rice [50] via a mechanism that inhibits cell wall loosening and cell growth. It has been shown that aphid feeding results in significantly altered transcripts associated with cell wall metabolism [51].

Our transcriptomic analysis also showed high variability between the individual biological replicates, which made the evaluation difficult and limited the number of significant DEGs. Reasons for this could be that the interaction of the herbivores with the plants varied from experiment to experiment, which could lead to different time courses of gene expression. In addition, the leaves sampled for the analyses may not have been in exactly the same physiological state because of different light and temperature gradients within the plant canopy. The difference in DEGs after caterpillar feeding between tansy plants that were first attacked by aphids and those that were not was minimal. Due to the fluctuations in gene expression, we cannot exclude the possibility that we have overseen some possible synergistic interactions between aphid and caterpillar attack.

### Transcriptome: synthases

Tansy has a large chemical diversity of terpenoids. It is therefore not surprising that we were able to detect many transcripts with high similarity to terpene synthases in the de novo assembled transcriptome of plant chemotype 3. The phylogenetic analysis of putative terpene synthase genes shows that the tansy genes belong to the known *TPS* subgroups in dicotyledonous plants. Seven genes in group *TPS-b* which comprises monoterpene synthases, 16 in group *TPS-a*, which is characteristic for sesquiterpene synthases. Furthermore, we could detect one putative gene in the *TPS-f* group, which includes linalool synthases. The high homology of individual *TPS* genes to known and enzymatically characterised *TPS* does not imply that the enzymes annotated in tansy have the same biosynthesis products [8, 52–54]. In order to make a conclusive statement about the enzyme activity of the putative *TPS*, we are aware that each gene must be expressed heterologously and subjected to biochemical function analysis [55–58].

## Conclusions

In conclusion, our work shows that there is a chemotype-specific response in plants to herbivore attack, and that this also extends to priming, i.e. the response of plants to a second herbivore attack by another species. This will be of relevance to researchers investigating plant chemical communication in the field. If neighbouring chemotypes in a field population respond differently to herbivory/dual herbivory (i.e. some chemotypes respond by emitting higher levels of VOCs than others), this could potentially have effects ranging from the individual level to the group level. Individuals of some chemotypes may respond more efficiently to herbivory stress than others, and in a group setting these “louder” chemotypes might influence the local insect community, including natural enemies of herbivores, and other neighbouring plants.

## Methods

### Plant and insect material

Tansy seeds were collected with the consent of the property owner on a field site in southern Germany (N 48°25′1.51“; E 11°46’1.19”), described earlier in [6] following the Convention on the Trade in Endangered Species of Wild Fauna and Flora. The parental plants were formally identified by S. Zytynska as *Tanacetum vulgare* (L.). Five different tansy genotypes were grown from seeds and propagated by cuttings in the greenhouse in 2015 (the same year the experiment was performed). Plants were grown to a size of approximately 30 cm high in order to fit the size limitations of the glass cuvette system. The volatile terpenoid pattern of each genotype was assessed using the leaf hexane extraction method as outlined in [6]. Nine clonal daughters of each genotype were generated (via splitting), with the six healthiest plants selected for use in the experiment to serve as biological replicates.

All aphids (*Metopeurum fuscoviride* Stroyan) were kept under greenhouse conditions (21.7 °C, 70% relative humidity, 16:8 h light:dark) at Dürnast Experimental Station, Technical University Munich, Freising, Germany, and were reared on chemotypes not used in the experiment. (for further rearing details see [35]). As a chewing herbivore, we chose *Spodoptera littoralis* Boisduval, the African cotton leafworm, as it is a generalist caterpillar that can cause massive trauma to plants, oftentimes completely stripping a plant of its foliage. It has been shown that specific VOCs are released in response to *S. littoralis* feeding on tansy, with a similar VOC profile emitted upon application of *S. littoralis* oral secretions [59]. First instar larvae of *S. littoralis* were reared on commercial lettuce leaves at room temperature. All larvae were starved for 24 h prior to application on the plants in order to ensure immediate feeding.

### Initial selection of terpenoid chemotypes

In order to select plants containing a wide range of compounds, we assessed the VOC profile of eight tansy plants using the hexane extraction method outlined in [6]. A total of 48 compounds were identified, and five chemotypes were assigned according to relative dominance of compounds (Fig. 1a). All plant chemotypes were propagated by splitting plants into nine daughter clones, which were further used as biological replicates as the chemotype is stable among clones [6].

### Experimental design

In the central experiment, five tansy “chemotypes” (Fig. 1a) with six biological replicates each, were exposed to caterpillars after they had been attacked or not attacked by aphids (Fig. 1b, six plant replicates per chemotype, three replicates not treated with aphids, three replicates treated with aphids, all replicates later treated with caterpillars). On day zero of the experiment five randomised tansy plants that were approximately 30 cm tall were placed into cuvette bases within the system described shown in Fig. S1, with one cuvette kept empty to provide an empty background for statistical normalisation. It is important to note that when handling aromatic plants extreme care must be taken to avoid jostling the leaves so as to minimise disruption of oil storage cavities (trichomes). An appropriate amount of time must have passed for the plant to “settle down” after events such as applying the herbivores and placing the plant in the system. A large glass bulb was then carefully placed over each plant. The plants were cultivated with a day-night change of 16/8 h at a light intensity of approximately 200 μmol photons m^− 2^ s^− 1^ (Agro Son-T 400 W, Philips, Hamburg, Germany) above the plants. Temperature varied from 23 °C at night to > 30 °C during the light phase. All plants were allowed to equilibrate for 24 h. On day 1 of the experiment at midday, 100 unwinged adult aphids were carefully applied to the aphid treatment plants. On day 4 of the experiment two second-instar *S. littoralis* larvae were then carefully applied to each plant. The experiment concluded on day 7. All *S. littoralis* larvae were collected and destroyed in accordance with Council Directive 2000/29/EC.

We sampled our two-stage experiment twice at two sampling times (day 4, day 7), to look at the effect of aphid infestation only. In the first half of the experiments, plants not attacked by aphids (no aphids, no caterpillars) were labelled “N”; and plants with only aphid treatment (aphids, no caterpillars) were labelled “A”. In the second half of the experiment, plants that had previously not been attacked by aphids but received caterpillars (no aphids, caterpillars) were labelled “C”, and aphid-treated plants that received caterpillars (both aphids and caterpillars) were labelled “B”. Figure 1a shows the VOC pattern of plants in treatment group N, i.e. the VOC emission of the different chemotypes when not attacked by a herbivore.

Leaf samples were collected from all plants first at day 4. We carefully avoided areas infested with aphids, and froze leaves immediately in liquid nitrogen, which were then stored at − 80 °C until further use. On day 7, all remaining leaves from all plants were harvested and immediately frozen in liquid nitrogen. Headspace volatiles were collected daily using mixed Tenax/Carbopack tubes (see Fig. 1b). All cuvettes were continuously monitored by PTR-ToF-MS in addition. Transcriptome analysis was performed on leaf material from plant chemotype 3.

### Real time analysis of emitted VOCs

On-line analysis of emitted volatiles was conducted using the multiple cuvette system [12] comprising of six whole plant cuvettes coupled to a commercial PTR-ToF-MS, Ionicon Analytik GmbH, Innsbruck, Austria). The cuvettes consist of a glass bulb atop a gas tight cylindrical stainless-steel base using inert Viton rings to seal the joint (for details see Jud et al. [12]). The base contains connections for gas and irrigation tubing, as well as electrical connections. The cuvettes were flushed with air drawn from the experimental hall via a rotary vane compressor (DLT 40, Gardner Denver Schopfheim GmbH, Schopfheim, Germany). Air flow was set to 18 L min^− 1^ from weeks one to three and reduced to 10 L min^− 1^ from the beginning of week four for the remaining duration of the experiment. Air flow (~ 120 mL min^− 1^) to the PTR-ToF-MS alternated between cuvettes, with switching time set to five minutes. The instrument was operated with an E/N of 115 Td (E = the electric field strength, N = the gas number density; 1 Td = 10^− 17^ V cm^2^; drift tube pressure = 2.2 mbar; drift voltage = 500 V, drift tube temperature = 60 °C). Throughout the experiments, the ions H_3_O·H_2_O^+^, O_2_^+^, and NO^+^ were kept below 10, 3, and 0.2% of the primary ions, respectively. The range of mass spectra was set to record up to *m/z 318*. The PTR-ToF-MS raw data were analysed using the routines described in [12, 60]. Data were analysed as described in [61]. Briefly, calculated signals in counts per second were normalised to account for differences in the absolute humidity of each cuvette, giving the signals in normalised counts per second (ncps).

### GC-MS analysis of VOCs from absorption tubes and hexane extraction

For the VOC emission analysis by GC-MS from the cuvette measurements, a series of sorbent tubes containing 40 mg Tenax TA /10 mg Carbopack (both obtained from Sigma-Aldrich, Taufkirchen, Germany [43];) were coupled to the flow of air drawn (0.1 L min^− 1^; collection period of 3 h around noon) from the outlet air of each cuvette on days four and seven (Fig. S1). After sampling, the Tenax/ Carbopack tubes were closed under gastight conditions and stored until analysis at 4 °C. For GC-MS analysis the tubes were desorbed using a thermo-desorption unit (TDU; Gerstel GmbH, Müllheim an der Ruhr, Germany) coupled to a gas chromatography mass-spectrometer (GC-MS; GC: 7890A, MS: 5975C inert XL MSD with a triple axis detector, both Agilent Technologies, Palo Alto, CA, USA). The CIS vaporisation inlet (cooled injection system; Gerstel) was set at − 50 °C. The TDU was heated to 290 °C from 37 °C at a rate of 280 °C min^− 1^. Samples were analysed splitlessly at a constant flow rate of helium at 1 mL min^− 1^. After 0.2 min the CIS was heated to 290 °C at a rate of 12 °C min^− 1^ and held for 2 min (method adapted from Ghirardo et al. 2012 [43]).

For the analysis of stored terpenoid compounds in leaves, hexane extracts were prepared from frozen leaf powder collected on days four and seven, and analysed by GC-MS. The extracts were prepared and analysed using the procedures and GC-MS methods as described in [6].

Identification and quantification of all VOCs was performed through comparison of obtained mass spectra with NIST 05 and Wiley library spectra, those of commercial standards (Sigma-Aldrich, Taufkirchen, Germany), and the Kovats retention index library [62].

### RNA extraction

On days 4 and 7, leaf tissue that had been badly damaged by *S. littoralis* or infested with *M. fuscoviride*, (however not containing any insect material) as well as control plants, was selected for analysis, with care being taken to avoid any aphids (*n* = 3 for each treatment group, with 12 replicates in total). Leaf material was ground under liquid nitrogen. Total RNA extraction was performed using the innuPREP Plant RNA Kit from Analytik Jena AG (Jena, Germany) according to the manufacturer’s instructions. RNA quality was confirmed using the Agilent 2100 Bioanalyzer with the RNA6000 Nano Lab Chip Kit (both Agilent Technologies, Palo Alto, CA, USA); the RNA Integrity Number (RIN) was between 7.5–8.

cDNA library construction and next-generation sequencing of transcripts were performed by Vertis Biotechnologie AG (Freising, Germany). Firstly, all samples were treated with DNase in order to remove any genomic DNA and then examined using a Shimadzu MultiNA microchip electrophoresis system (Shimadzu, Japan). Poly(A) + RNA was then isolated from the total RNA samples. First-strand cDNA was synthesised using an N6 randomised primer. Following fragmentation, the Illumina TruSeq sequencing adapters were ligated to the 5′ and 3′ ends of the cDNA fragments in a strand-specific manner. Finally, the cDNA was amplified with PCR (14–15 cycles) using a proof-reading enzyme. cDNA was then pooled in approximately equimolar amounts, and eluted from a preparative agarose gel in the size range of 350–500 bp. An aliquot of the size fractionated cDNA pool was analysed with capillary electrophoresis. The NGS library pool was paired-end sequenced with an Illumina NextSeq 500 system using 150 bp (read 1) and 150 bp (read 2) read length and a ‘HIGH 300’ sequencing kit.

RNA-Seq data were assembled to transcript sequences applying two assemblers, the TRINITY platform [63] using default parameters and the entire read set, and Bridger [64] applying a kmer series (K = 25,27,31) for combined replicates of each of the four samples separately. Resulting transcripts were merged into a consensus transcriptome assembly using the Evigene pipeline (http://arthropods.eugenes.org/EvidentialGene/) with default settings. Subsequently, reported transcripts were filtered for coding potential by Transdecoder v2.0.1 [65] including pfam searches and homology results. In total, 110,253 transcript/gene loci represented by 180,353 alternative splice variants were obtained. All transcripts were compared to an invertebrate protein dataset of NCBI. Sequences for which their top scoring hit matched a plant protein were retained in the final tansy transcriptome. This approach removed 69,613 transcripts with no protein homology and 16,578 insect related transcripts, resulting in 52,765 tansy transcript loci and 94,162 splice variants. Coding ORFs of the transcripts were annotated applying the AHRD pipeline (https://github.com/groupschoof/AHRD) and using default settings for the Interpro and Pfam searches, and homology comparisons to the *Arabidopsis thaliana*, Swissprot and Trembl databases.

Differential gene expression (DEG) analysis was performed using the R package *‘edgeR*’ v3.26 [66] applying a standard protocol. Briefly, salmon in quasi-mapping mode was applied to the tansy transcriptome for each sample and replicate to obtain a digital count matrix. To reduce data complexity and avoid overly stringent multiple hypothesis correction, we proceeded with mean counts of alternative splice variants for each transcript locus. Transcripts with low counts were filtered by the function ‘*filterByExpr*’ and DEGs were derived by GLM (generalised linear models) method as provided in the *edgeR* package. After filtering, loci selected for DEG analysis totalled 31,928 loci. Four contrasts were analysed: A-N (with and without aphid treatment, *(a)* ‘aphid effect’), B-A and C-N (caterpillar treatment with and without pre-inoculation by aphids, *(ca)* and *(c)* respectively), and the combined contrast [(B-A) - (C-N)], *(d)*, the difference in response to caterpillars in the presence or absence of a pre-treatment by aphids; see Fig. S3.

Related TPS protein sequences from were obtained from NCBI, with an E-value cutoff of 1 × 10^− 5^. Protein sequences were aligned using MUSCLE [67]; poorly aligned positions were removed using Gblocks [68] and MaxAlign [69]. The maximum-likelihood phylogenetic tree was generated using RAxML [70] with 1000 bootstrap replications, and was drawn in Dendroscope [71].

### Accession numbers

Raw and processed RNA-Seq data have been deposited at the NCBI Sequence Read Archive (SRA) with the BioProject number PRJNA646340.

### Statistics

The metabolic profiles of all plant chemotypes and different treatments were analysed using MetaboAnalyst 3.0 [72]. Statistical analyses were performed using MetaboAnalyst 3.0 and SigmaPlot Version14 [73].

## Supplementary Information

**Additional file 1:** **Figure S1:** Schematic of cuvette platform and experimental setup.

**Additional file 2:** **Figure S2**: Two-tailed t-test analysis of summed concentrations of all VOCS (measured in hexane extracts) across chemotypes per treatment. A significant difference was found between treatment groups B and N (t-value = − 3.659, df = 4, *P* = 0.022). N: no aphid, no caterpillar, leaf material harvested on day 4; A: aphid, no caterpillar, leaf material harvested on day 7; C: no aphid, caterpillar, leaf material harvested on day 4; B: both aphid and caterpillar, leaf material harvested on day 7.

**Additional file 3:** **Figure S3:** Visual representation of treatment groups for transcriptome analysis.

**Additional file 4:** **Figure S4:** Phylogenetic tree of *TPS* genes obtained from tansy and other related species. *TPS* subfamilies are coloured as follows, blue: green: *TPS-a, TPS-b*, yellow: *TPS-f*.

**Additional file 5:** **Table S1:** Compounds identified in hexane and SBSE extraction analysis methods.

**Additional file 6:** **Table S2**: Mean concentration of compounds of each plant chemotype found in hexane extractions at sampling days 4 and 7. **Table S3**: Mean VOC emission rates of each plant chemotype at sampling days 1–4 and 4–7. **Table S4**: Mean VOC emission rates of representative masses (m/z) measured using PTR-ToF-MS of different each plant chemotype at sampling days 1–4 and 4–7.

**Additional file 7:** **Table S5:** List of differentially expressed genes.

**Additional file 8:** **Table S6:** List of differentially expressed genes associated with defence responses.

## Data Availability

The raw and processed transcriptomic datasets supporting the conclusions of this article have been deposited at the NCBI Sequence Read Archive (SRA) under bioproject number PRJNA646340 accessible at https://www.ncbi.nlm.nih.gov/bioproject/PRJNA646340 or https://www.ncbi.nlm.nih.gov/sra/?term=PRJNA646340.The other datasets supporting the conclusion of this article are included within the article (and its additional files).

## Abbreviations

VOCs: Volatile organic compounds
GC-MS: Gas chromatography mass spectrometry
PTR-ToF-MS: Proton transfer reaction time-of-flight mass spectrometry
TPS: Terpene synthase
DEGs: Differentially expressed genes
SAR: Systemic acquired resistance
DMNT: Dimethylnonatriene
RNA-seq: RNA next generation sequencing
