# Supplementary figures and images for: Under fire-simultaneous volatilome and transcriptome analysis unravels fine-scale responses of tansy chemotypes to dual herbivore attack
Source: BMC Plant Biol. 2020 Dec 9;20:551. doi: 10.1186/s12870-020-02745-1 (PMC7724791; doi:10.1186/s12870-020-02745-1)

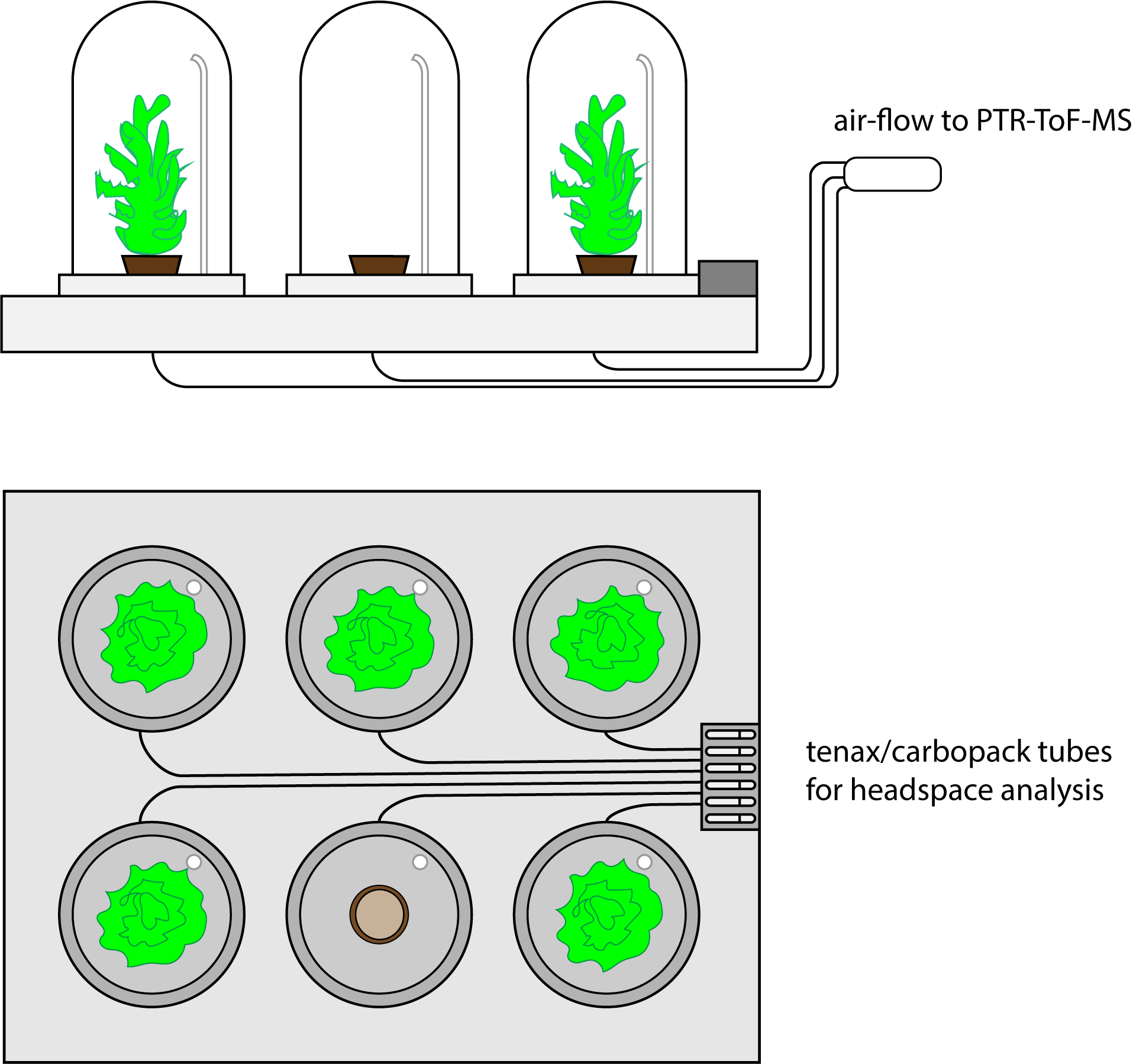

Supplement: Supplementary file 1 — Additional file 1: Figure S1: Schematic of cuvette platform and experimental setup. [file 12870_2020_2745_MOESM1_ESM.tif]

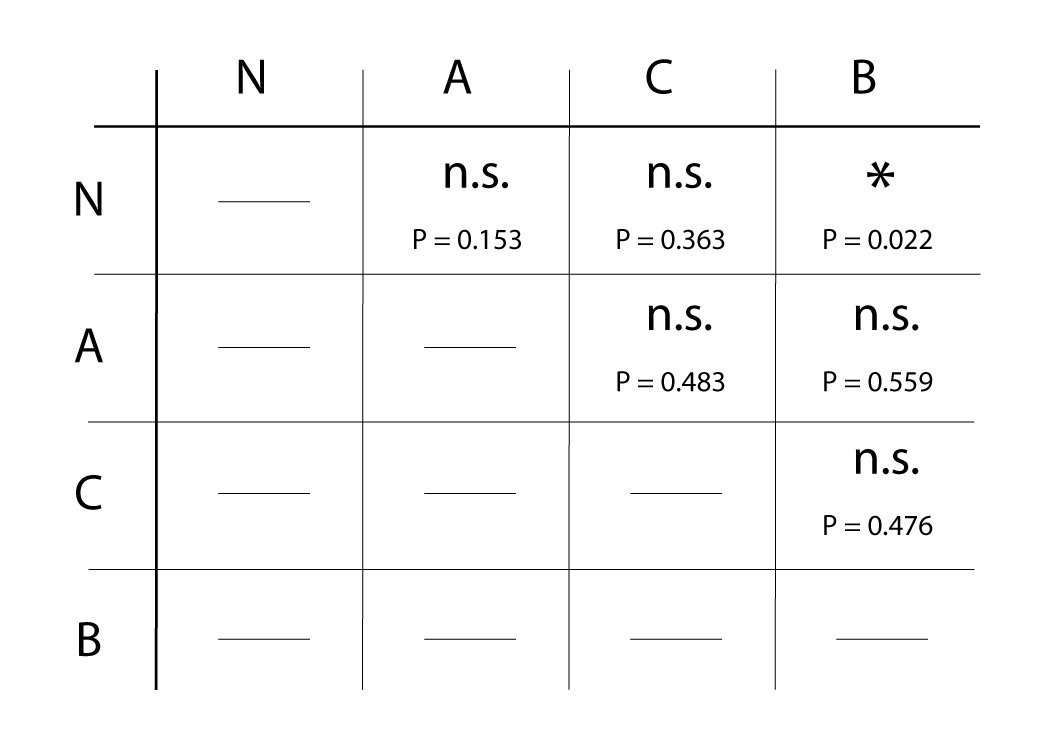

Supplement: Supplementary file 2 — Additional file 2: Figure S2: Two-tailed t-test analysis of summed concentrations of all VOCS (measured in hexane extracts) across chemotypes per treatment. A significant difference was found between treatment groups B and N (t-value = − 3.659, df = 4, P = 0.022). N: no aphid, no caterpillar, leaf material harvested on day 4; A: aphid, no caterpillar, leaf material harvested on day 7; C: no aphid, caterpillar, leaf material harvested on day 4; B: both aphid and caterpillar, leaf material harvested on day 7. [file 12870_2020_2745_MOESM2_ESM.tif]

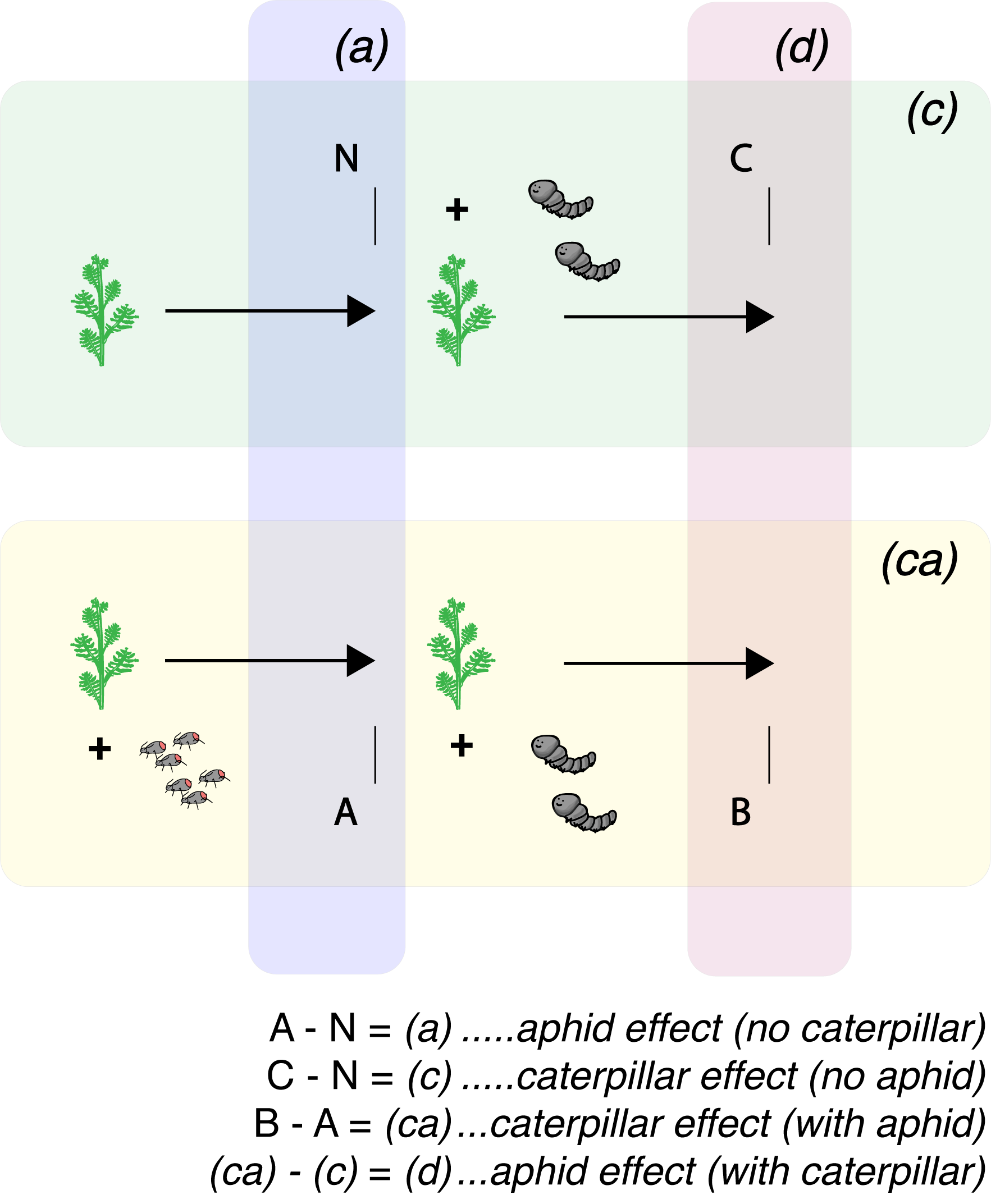

Supplement: Supplementary file 3 — Additional file 3: Figure S3: Visual representation of treatment groups for transcriptome analysis. [file 12870_2020_2745_MOESM3_ESM.tif]

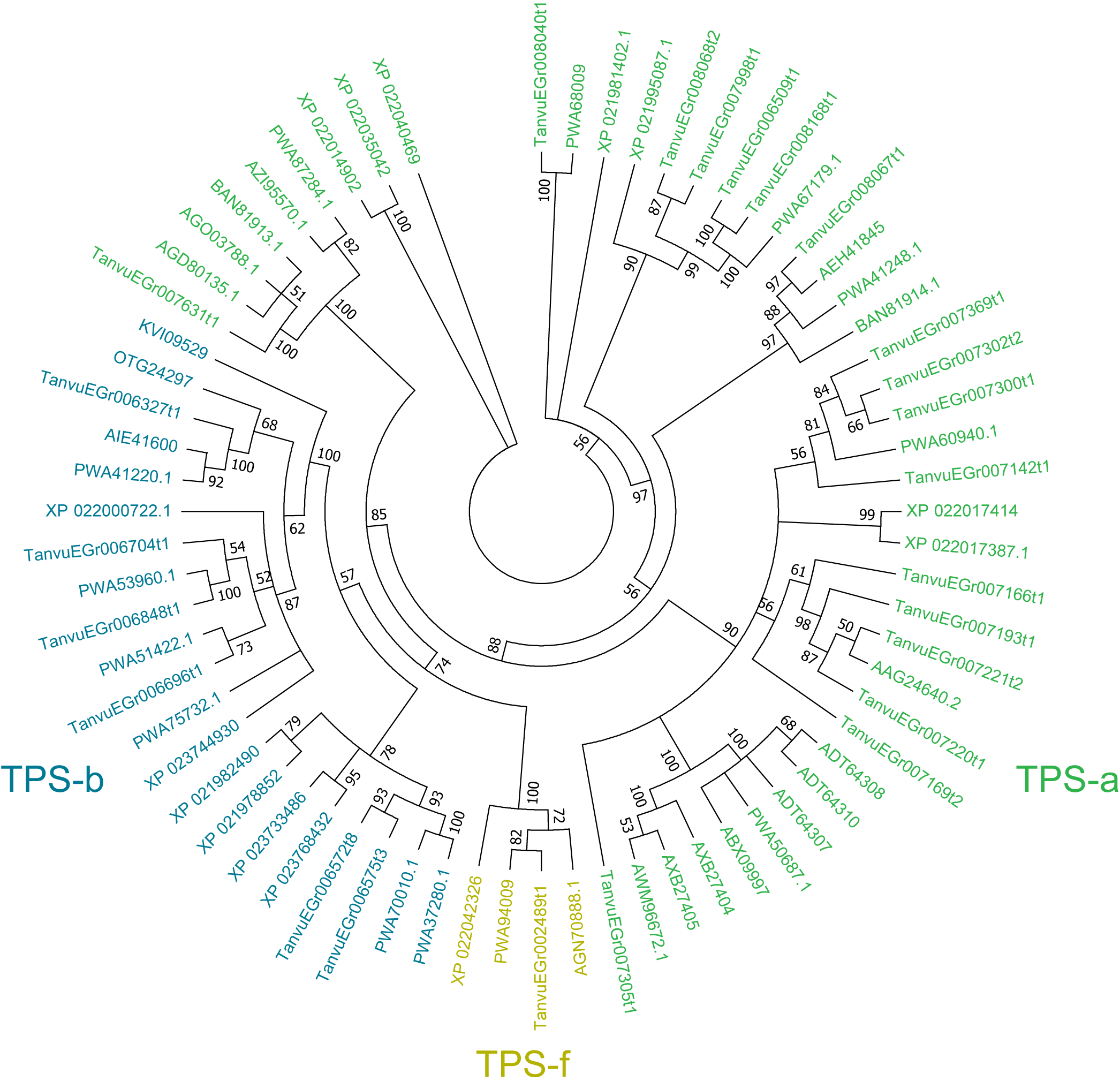

Supplement: Supplementary file 4 — Additional file 4: Figure S4: Phylogenetic tree of TPS genes obtained from tansy and other related species. TPS subfamilies are coloured as follows, blue: green: TPS-a, TPS-b, yellow: TPS-f. [file 12870_2020_2745_MOESM4_ESM.tif]

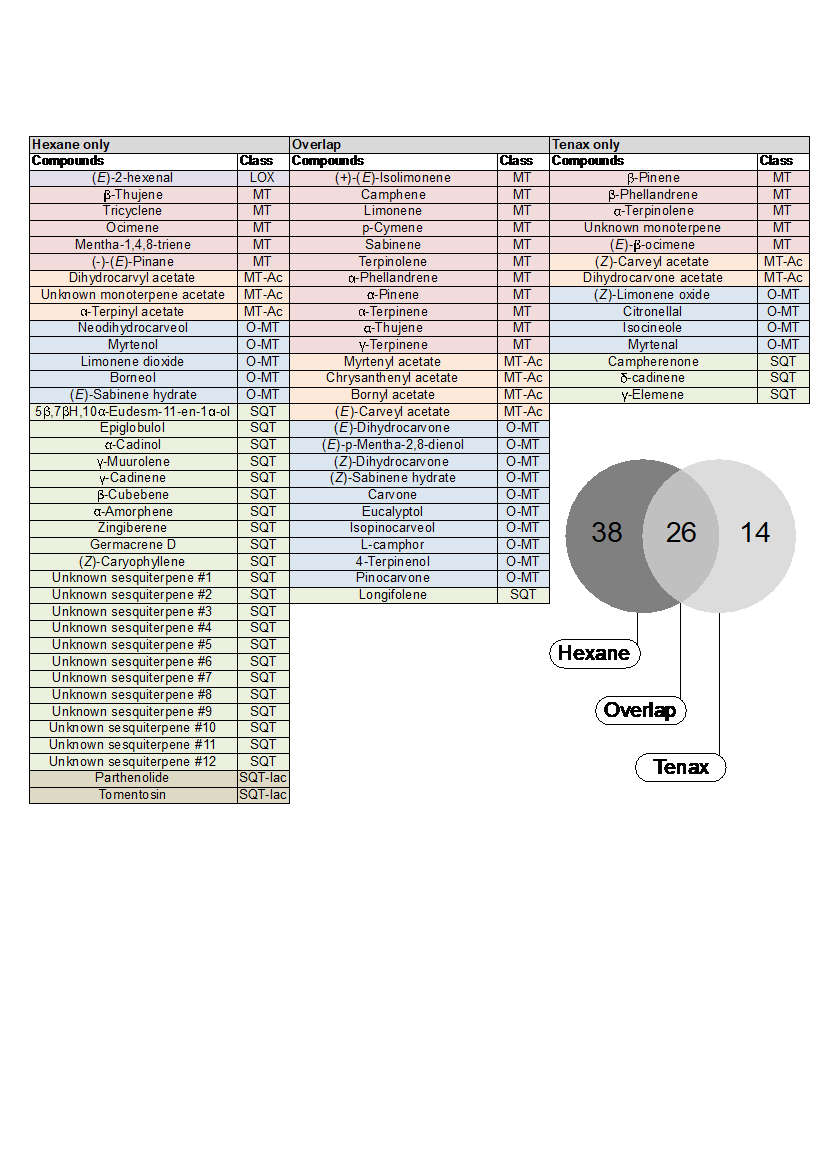

Supplement: Supplementary file 5 — Additional file 5: Table S1: Compounds identified in hexane and SBSE extraction analysis methods. [file 12870_2020_2745_MOESM5_ESM.tif]
